# Intersexuality and the Cricket Frog Decline: Historic and Geographic Trends

**DOI:** 10.1289/ehp.7276

**Published:** 2004-12-07

**Authors:** Amy L. Reeder, Marilyn O. Ruiz, Allan Pessier, Lauren E. Brown, Jeffrey M. Levengood, Christopher A. Phillips, Matthew B. Wheeler, Richard E. Warner, Val R. Beasley

**Affiliations:** ^1^Department of Animal Sciences, and; ^2^Department of Veterinary Pathobiology, University of Illinois at Urbana-Champaign, Champaign, Illinois, USA; ^3^University of Illinois Zoological Pathology Program, Loyola University Medical Center, Maywood, Illinois, USA; ^4^Department of Biological Sciences, Illinois State University, Normal, Illinois, USA; ^5^Department of Veterinary Biosciences, and; ^6^Department of Natural Resources and Environmental Sciences, University of Illinois at Urbana-Champaign, Champaign, Illinois, USA; ^7^Center for Biodiversity, Illinois Natural History Survey, Champaign, Illinois, USA

**Keywords:** *Acris crepitans*, amphibian, cricket frogs, endocrine disruption, environmental contaminants, Illinois, intersexuality

## Abstract

Exposure to anthropogenic endocrine disruptors has been listed as one of several potential causes of amphibian declines in recent years. We examined gonads of 814 cricket frogs (*Acris crepitans*) collected in Illinois and deposited in museum collections to elucidate relationships between the decline of this species in Illinois and the spatial and temporal distribution of individuals with intersex gonads. Compared with the preorganochlorine era studied (1852–1929), the percentage of intersex cricket frogs increased during the period of industrial growth and initial uses of polychlorinated biphenyls (PCBs) (1930–1945), was highest during the greatest manufacture and use of *p*,*p*-dichlorodiphenyltrichloroethane (DDT) and PCBs (1946–1959), began declining with the increase in public concern and environmental regulations that reduced and then prevented sales of DDT in the United States (1960–1979), and continued to decline through the period of gradual reductions in environmental residues of organochlorine pesticides and PCBs in the midwestern United States (1980–2001). The proportion of intersex individuals among those frogs was highest in the heavily industrialized and urbanized northeastern portion of Illinois, intermediate in the intensively farmed central and northwestern areas, and lowest in the less intensively managed and ecologically more diverse southern part of the state. Records of deposits of cricket frog specimens into museum collections indicate a marked reduction in numbers from northeastern Illinois in recent decades. These findings are consistent with the hypothesis that endocrine disruption contributed to the decline of cricket frogs in Illinois.

Amphibian declines have been documented in many parts of the world (Alford et al. 2001; Blaustein and Wake 1990; Houlahan et al. 2000; Tyler 1991), which is of concern because these species are important grazers, prey species, and predators in aquatic and terrestrial ecosystems and serve as valuable sentinels of ecologic integrity. For many amphibian species, including the cricket frog *Acris crepitans*, the causes of declines are unclear. This species is indigenous to the eastern half of the United States and has experienced a marked decline in portions of its range in the last 25 years (Brodman and Kilmurry 1998; Caspar 1998; Jung 1993; Mierzwa 1998; Minton 1998; Moriarity 1998; Mossman et al. 1998). In 1961, it was the most common amphibian in Illinois (Smith 1961), and it is still abundant in southern Illinois. However, cricket frogs were rarely encountered in amphibian surveys conducted in portions of northern Illinois in recent decades (Ludwig et al. 1992; Vogt 1981; Phillips CA, Brown LE, personal observation).

A variety of industrial compounds and by-products disrupt endocrine function, and exposure to such chemicals may contribute to amphibian declines. Polychlorinated biphenyls (PCBs), polychlorinated dibenzofurans (PCDFs), and polychlorinated dibenzo-*p*-dioxins (PCDDs) may produce estrogenic, antiestrogenic, and antiandrogenic effects (Jansen et al. 1993; Krishnan and Safe 1993; Li and Hansen 1996; Li et al. 1994; Malby et al. 1992). For example, PCBs may affect sexual differentiation in the slider turtle *Trachemys scripta* (Bergeron et al. 1994), various frogs (Qin et al. 2003), and fish (Matta et al. 1998). Moreover, our laboratory associated exposures of cricket frog tadpoles to antiestrogenic PCBs and PCDFs with marked masculinization of sex ratios (Reeder et al. 1998). In addition, polycyclic aromatic hydrocarbons (PAHs) from coal tar and smoke from combustion of coal, oil, gas, wood, and garbage are widely disseminated endocrine disruptors (Chaloupka et al. 1992, 1993; Machala et al. 2001; ATSDR 1995; Thomas and Budiantara 1995).

Pesticides now banned from the market and compounds still sold can adversely affect animal reproduction. For example, the organochlorine pesticide *p*,*p*-dichlorodiphenyl-trichloroethane (DDT) and its metabolites/ environmental products have demasculinized birds (e.g., gulls, *Larus californicus* and *L. occidentalis*), reptiles (*Alligator mississippiensis*), and fish (Baatrup and Junge 2001; Fry and Toone 1981; Guillette et al. 1994, 1995; Metcalfe et al. 2000). Also, DDT induced vitellogenin production in male African clawed frogs (*Xenopus laevis*) and slider turtles (*T. scripta*), and the organochlorine insecticides toxaphene and dieldrin induced this protein in male *X. laevis* (Palmer and Palmer 1995; Palmer et al. 1998). Furthermore, there is concern that the widely used herbicide atrazine may impair reproduction and/or development of amphibians. At high concentrations, atrazine decreased growth in gray treefrogs (*Hyla versicolor*) and increased time to metamorphosis in *X. laevis* (Diana et al. 2000; Sullivan and Spence 2003). At much lower concentrations, atrazine in combination with nitrate reduced growth of *X. laevis* (Sullivan and Spence 2003). Hayes et al. (2002, 2003) associated hermaphrodism and gonadal dysgenesis in amphibians with very low aquatic concentrations of atrazine (0.1 μg/L), whereas Carr et al. (2003) observed significantly increased intersexuality only at higher concentrations (25 μg/L).

Although it is apparent that exposure to anthropogenic compounds may harm amphibians through changes in functional sex ratios, reduced size at metamorphosis, and delayed maturation, the distribution of intersex frogs geographically and historically remains to be characterized. In this study, we examined gonads of *A. crepitans* from museum specimens to elucidate relationships between the decline of this species and the temporal and spatial occurrence of intersexuality.

## Materials and Methods

Natural history museums are valuable resources for estimations of species distributions and health status over time (Shaffer et al. 1998). We examined specimen records from 16 museums to determine where and when cricket frogs were collected in Illinois. To determine whether cricket frogs were not available because collecting was not conducted, we compared cricket frog records with those of all anuran collections from the state. Our rationale was that scientists collecting anurans and placing them in museums as voucher specimens would not consistently preclude cricket frogs.

**Figure 1:**
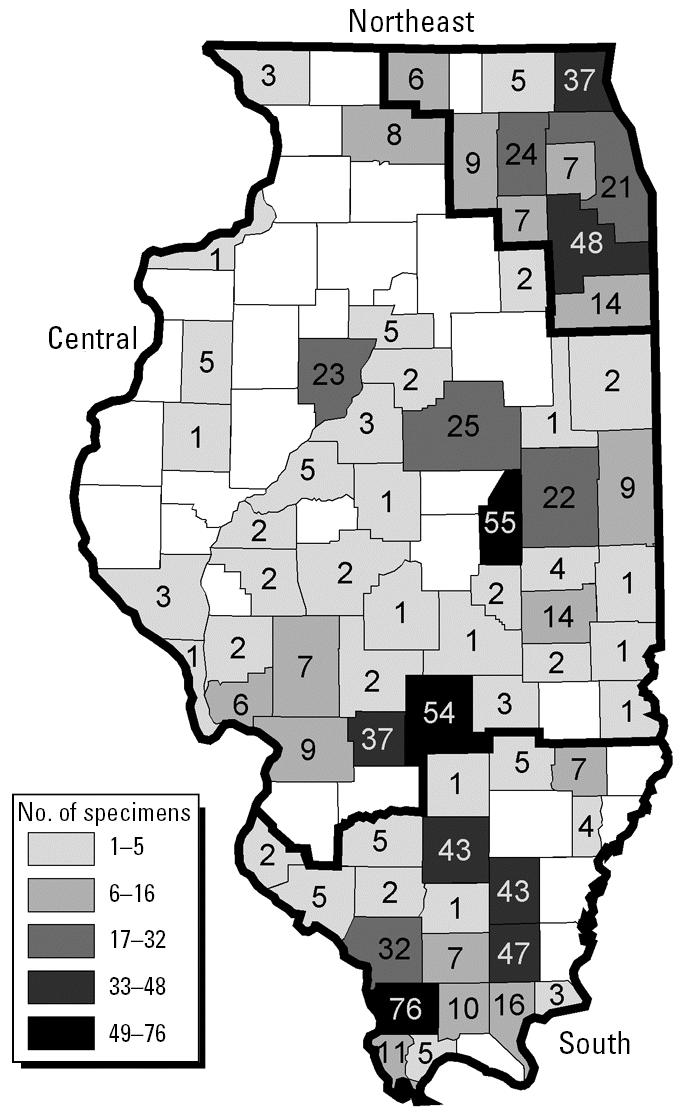
Number by county of cricket frogs (*n* = 814) collected in Illinois from 1852 to 1996 and examined for gonadal sex determination.

Museum specimens from throughout Illinois (Figure 1) were examined to compare cricket frog gonadal sex in three regions of the state during five time periods. The three regions are distinguished by human population density and physiographic characteristics. The northeast region includes 11 counties with high human population densities in and surrounding the Chicago metropolitan area. The central band of 66 counties, which was formerly largely prairie, is dominated by low topographic relief, fertile soils, intensive maize and soy agriculture, and low human population density. The southernmost region includes 25 counties with mixed crops, pastures, and wooded hills as well as low human population density. The five time periods studied included *a*) a preorganochlorine period (1852–1929); *b*) an era of PCB use and industrialization that predates use of DDT (1930–1945); *c*) a period of rapidly increasing DDT use and further industrialization (1946–1959); *d* ) a period of declining use and then a ban on sales of DDT as well as initial measures to limit pollution from industries (1960–1979); and *e*) a period associated with a substantial decline in environmental residues of organochlorine insecticides and other persistent halogenated organic air and water pollutants in the Midwest (1980–2001).

The gonads of 814 cricket frogs were examined *in situ* with a dissecting microscope to identify sex. Because there is no evidence that testicular tissue develops within the female tract, females were identified by the presence of oocytes and excluded from further analysis. Testes, intersex gonads, and poorly differentiated gonads were removed for histologic examination. When museums limited the proportion of cricket frogs from which gonads could be taken, we used a random numbers table to select a subset for histologic study. Collection dates, collecting localities, and notes from museum catalogs were recorded. Gonads were immersed in 70% ethanol, embedded in paraffin, and sectioned at 5 μm. Depending on gonad size, longitudinal sections were obtained beginning 60–150 μm from the outer edge. Two or three sections 60–120 μm apart were selected for processing. Slides were stained with hematoxylin and eosin and examined with a light microscope for the presence of sperm production and oocytes. When oocytes were present within testicular tissue, they were relatively few in number, but each was several-fold larger than the spermatic ducts and thus took up a substantial percentage of testicular volume, making them easy to identify.

We used Systat 10.2 (SPSS, Inc., Chicago, IL) to construct contingency tables and perform Pearson chi-square analyses comparing proportions of male, female, and intersex individuals among the five time periods and three areas of Illinois. Further analysis incorporating temporal and spatial dimensions simultaneously was not possible because of the limited numbers of intersex specimens at some time points. Specifically, tables based on all five time periods and three regions broken down by sex would have included cells with no data. The null hypotheses tested whether the proportion of male, intersex, and female frogs is the same for frogs captured during the five time periods and in the three areas of the state. We used the 5% significance level as the indicator of a statistically significant association.

## Results

**Figure 2:**
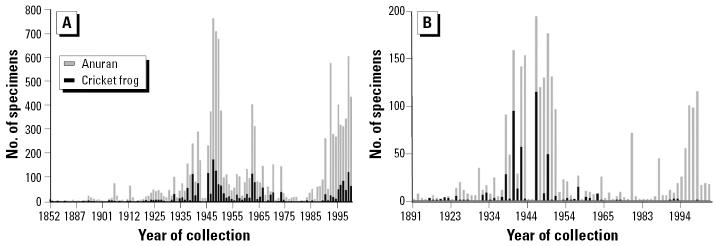
(*A*) Numbers of cricket frog specimens from Illinois deposited in museum collections relative to numbers of other anurans in museums collected in the state from 1852 to 2001. (*B*) Numbers of cricket frog specimens from northeastern Illinois deposited in museum collections relative to numbers of other anurans in museums collected in that region from 1852 to 2001.

**Figure 3:**
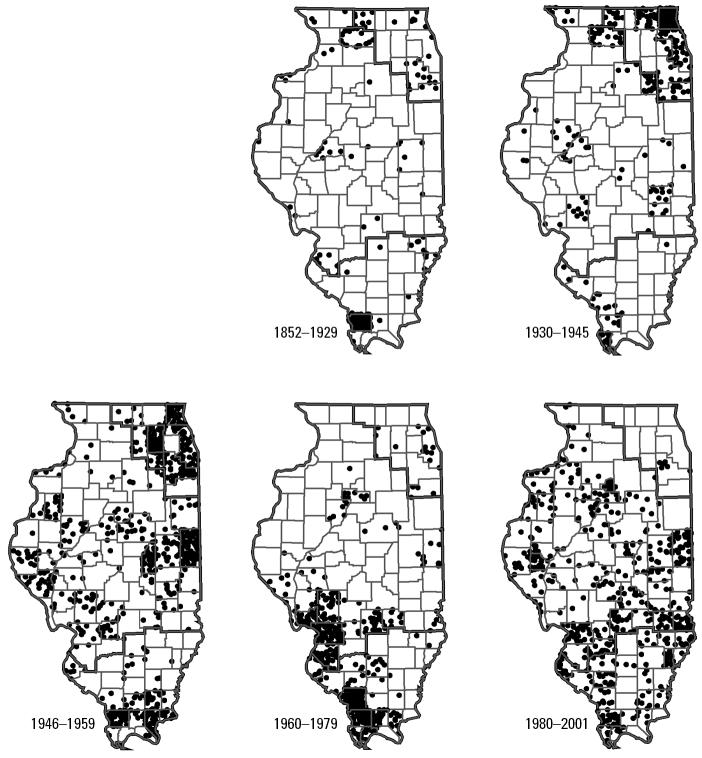
Distributions of total numbers of Illinois cricket frogs in museum collections for the five time periods. Each circle represents one museum specimen from that county.

Records for all anurans totaled 12,661 specimens. Of these, 2,570 (20%) were *A. crepitans*, the frog species most often collected in Illinois. Years of collection ranged from 1852 to 2001. A trend of increasing numbers of frogs collected started in the late 1930s; there was a marked reduction during World War II, and then the rate of collecting markedly increased through the mid-1950s. Numbers of individual anurans collected declined sharply in the late 1950s, increased during the mid-1960s, and declined during the 1970s and 1980s. The numbers of frogs collected in Illinois have since increased. Cricket frog numbers were largely proportional to those of other anurans (Figure 2A). Other frog and toad species have been collected from the Chicago region in proportion to other regions of the state since 1960; however, few cricket frogs were obtained from that area in the same time frame (Figure 2B). Moreover, from 1980 to 2001, cricket frog collections remained high in the central and southern regions but declined even further around Chicago (Figure 3).

**Figure 4:**
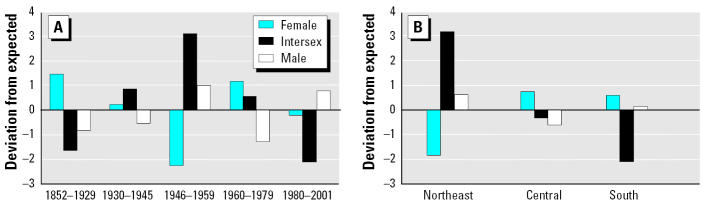
Deviations of observed from expected values of cricket frog sex (*A*) by time period and (*B*) by region. Expected values were determined from the overall data set using the chi-square test.

Intersexuality (hermaphrodism) was manifested in two forms: most intersex frogs (*n* = 37) had an ovotestis where proportionately large ova were present within testicular tissue, and a few (*n* = 6) had a complete testis and complete ovary. The proportions of specimens in each gonadal sex class differed significantly among the geographical regions in Illinois (χ^2^ = 20.2, 4 df, *p* < 0.001; Table 1, Figure 4A). Notably, in the urbanized northeastern portion of the state, the proportion of frogs that were intersex was much greater than in other areas, and the proportion of females was smaller than elsewhere. In southern Illinois, where agriculture and urbanization are least intensive, the proportion of intersex individuals was considerably smaller than in the other regions.

The proportion of specimens in each gonadal sex class differed markedly among the time periods of collection (χ^2^ = 31.1, 8 df, *p* < 0.001; Table 1, Figure 4B). From 1930 to 1945, the percentage of intersex individuals was notably increased, and from 1946 to 1959 it was greater than during any other time frame examined. Also, during 1946–1959, the proportion of females was reduced. During the most recent period, the proportion of intersexes was lowest of any period except for 1852–1929. In the 1990s, however, few cricket frogs were available from areas that previously had the most elevated intersex rates.

## Discussion

Environmental contamination probably accounted for the historical and geographical trends in gonadal sex in Illinois cricket frogs and likely contributed to the decline of the species. In this research, it would not have been productive to assay contaminants in tissues of the frogs because of their small size, and because frogs are individually tagged but stored together in jars of fixative with conspecific individuals, which would enable postmortem cross-contamination. Moreover, such use would consume the specimens, preventing any future examination for other research aims. Also, it would not be meaningful to assay contaminant mixtures at sites today because they would no longer be representative of what was present when the frogs were collected.

The absence of cricket frogs from northeastern Illinois in museum collections is consistent with reports indicating virtual disappearance of cricket frogs from this area (Ludwig et al. 1992; Phillips CA, Brown LE, personal observation; Vogt 1981). Heavy industrialization from the 1930s through the 1950s was accompanied by major releases of combustion products and organochlorine contaminants. Smokes contain abundant mixtures of PAHs that adsorb to particulates in air, soil, water, and sediment (Mumtaz and George 1995). Like coplanar PCDDs and similar organochlorines, some PAHs can act as antiestrogens (Chaloupka et al. 1992). By the 1930s, PCBs were being commercially produced (Hansen 1987), and coplanar PCBs and structurally similar PCDDs and PCDFs are potent antiestrogens. Thus, intensive industrial smoke emissions and commercial PCB production and widespread use coincided with the increase in the proportion of intersex cricket frogs. A possible outcome of exposure to various PAH and organochlorine antiestrogens is masculinization of juvenile cricket frogs and skewed sex ratios, as noted in the Chicago region in this study, which is similar to what we found previously at a hazardous waste site at Crab Orchard National Wildlife Refuge, where cricket frogs were contaminated with coplanar PCBs and PCDFs (Reeder et al. 1998).

The greatest proportions of intersex in cricket frogs of Illinois during 1946–1959 corresponded with a rapid increase in use of DDT in the United States (Mellanby 1992; U.S. Army Service Forces 1946). Large-scale DDT applications in Illinois for mosquito control began in 1945, followed by agricultural use in 1946. Production of DDT in the United States was greatest in 1959. Reduced prevalence of intersex in cricket frogs from 1960 to 1976 coincided with decreased use and the subsequent ban of most uses of DDT in the United States in 1972. Although exposure of larval tiger salamanders (*Ambystoma tigrinum*) to *p*,*p*-DDE (*p*,*p*-dichlorodiphenyl-dichloroethylene) stimulated growth of Mullerian ducts consistent with estrogenicity, exposure to technical-grade DDT had a paradoxical antiestrogenic effect (Clark et al. 1998). Thus, if cricket frogs responded to DDT exposure as did *A. triginum*, its use could have contributed to the concurrent decrease in female and increase in intersex cricket frogs during 1946–1959.

Atrazine was first marketed as a broadleaf herbicide for maize production in 1959, and use rapidly expanded. By 1993, the Midwest states of Illinois, Iowa, Nebraska, and Indiana accounted for 43% of the total amount of atrazine applied in the United States (Atrazine Ecological Risk Assessment Panel 1995), and it is still widely applied in the region. Based on findings of intersexuality at very low atrazine concentrations, Hayes et al. (2002, 2003) concluded that the widespread use of atrazine may have been a significant factor in amphibian declines. However, a recent study (Carr et al. 2003) indicated a higher threshold for atrazine-induced intersexuality in frogs. Additional research is needed to resolve this issue. Our study demonstrates that endocrine disruption and intersexuality were present in cricket frogs long before the advent of atrazine. However, the possibility that atrazine is one of the endocrine disruptors that contributed to the decline of cricket frogs and impedes expansion of its populations in central and northern Illinois is not ruled out by our findings.

Although we observed a decrease in intersex cricket frogs from Illinois after 1946–1959, McCallum and Trauth (2003) noted a progressive increase in the proportion of cricket frogs in Arkansas with external developmental abnormalities during four time periods from 1957 to 2000. Johnson et al. (2003) reported an increased prevalence of parasite-induced malformations by the trematode *Ribeiroia* from 1946 to 2002. Different patterns of contaminant and trematode exposures, and responses of germinal gonadal versus somatic cells to teratogenic stimuli may be responsible for the contrasting trends. Collection efforts and methods were not recorded in the catalogs and would have varied over time. Records typically were limited to the collector’s name and the location and date of acquisition in the field. Thus, bias that favored or limited collection of hermaphroditic cricket frogs cannot be completely ruled out. However, we have identified no basis for bias that would influence the likelihood of collecting intersex individuals. Hermaphroditic cricket frogs were collected by a wide range of investigators, and multiple hermaphrodites were found in the collections of many museums. Moreover, the museum records include no mention of behavioral or physical variation for any of the hermaphroditic specimens, and the collectors reported no knowledge of hermaphrodism. Finally, hermaphroditic individuals of this species can be identified only through gross dissection and histopathologic studies, which were not undertaken on these frogs prior to our research.

The geographic distribution of both endocrine disruption (intersexuality) and the decline of cricket frogs were congruent. The observed decline was evident after a period of sustained endocrine disruption, as indicated by a large increase in prevalence of intersex gonads and masculinization of the population. A plausible explanation for these observations is that exposures to antiestrogenic PAHs, PCBs, PCDFs, PCDDs, and DDT caused endocrine disruption, and this contributed to the virtual disappearance of cricket frogs from the Chicago region. The intersex prevalence in cricket frogs in recent years is low and may represent a near-baseline condition. A suite of endocrine-disrupting organochlorine contaminants persists in soils and waters of the Midwest, but at substantially reduced levels compared with earlier decades (Abramowicz 1990). However, we cannot conclude that the era of endocrine disruption in cricket frogs has come to an end, because in areas with the most severe decline in populations and most severe endocrine disruption historically, numbers of remaining cricket frogs are now insufficient to permit sampling.

## Figures and Tables

**Table 1:** Numbers (percentages) of cricket frog specimens by gonadal sex and region and by gonadal sex and time period.

	Female	Intersex	Male	Total observed
Region				
Northeast	57 (32.8)	19 (10.9)	98 (56.3)	174
Central	146 (44.7)	16 (4.9)	165 (50.5)	327
South	138 (44.1)	8 (2.6)	167 (53.4)	313
Total	341 (41.9)	43 (5.3)	430 (52.8)	814
Time period				
1852–1929	44 (52.4)	1 (1.2)	39 (46.4)	84
1930–1945	35 (43.8)	6 (7.5)	39 (48.8)	80
1946–1959	46 (30.1)	17 (11.1)	90 (58.8)	153
1960–1979	76 (48.1)	10 (6.3)	72 (45.6)	158
1980–1996	140 (41.3)	9 (2.7)	190 (56.1)	339
Total	341 (41.9)	43 (5.3)	430 (52.8)	814
